# Umbilical Cord Mesenchymal Stem Cell Secretome: A Potential Regulator of B Cells in Systemic Lupus Erythematosus

**DOI:** 10.3390/ijms252312515

**Published:** 2024-11-21

**Authors:** Adelina Yordanova, Mariana Ivanova, Kalina Tumangelova-Yuzeir, Alexander Angelov, Stanimir Kyurkchiev, Kalina Belemezova, Ekaterina Kurteva, Dobroslav Kyurkchiev, Ekaterina Ivanova-Todorova

**Affiliations:** 1 University Hospital St. Ivan Rilski, Laboratory of Clinical Immunology, Department of Clinical Immunology, Medical Faculty, Medical University of Sofia, 15 Akademik Iv. E. Geshov Blvd., 1431 Sofia, Bulgaria; ktuzeir@medfac.mu-sofia.bg (K.T.-Y.); e.kurteva@medfac.mu-sofia.bg (E.K.); dkyurkchiev@medfac.mu-sofia.bg (D.K.); etodorova@medfac.mu-sofia.bg (E.I.-T.); 2 University Hospital St. Ivan Rilski, Clinic of Rheumatology, Department of Rheumatology, Medical Faculty, Medical University of Sofia, 13 Urvich St., 1612 Sofia, Bulgaria; mariana_ig@abv.bg (M.I.); doc.a.angelov@gmail.com (A.A.); 3 Tissue Bank BulGen, 1330 Sofia, Bulgaria; stanimirkyurkchiev@gmail.com (S.K.); kalina.belemezova@gmail.com (K.B.)

**Keywords:** B lymphocytes, B-cell activating factor receptor, mesenchymal stem cells, systemic lupus erythematosus

## Abstract

Autoimmune diseases represent a severe personal and healthcare problem that seeks novel therapeutic solutions. Mesenchymal stem cells (MSCs) are multipotent cells with interesting cell biology and promising therapeutic potential. The immunoregulatory effects of secretory factors produced by umbilical cord mesenchymal stem cells (UC-MSCs) were assessed on B lymphocytes from 17 patients with systemic lupus erythematosus (SLE), as defined by the 2019 European Alliance of Associations for Rheumatology (EULAR)/American College of Rheumatology (ACR) classification criteria for SLE, and 10 healthy volunteers (HVs). Peripheral blood mononuclear cells (PBMCs) from patients and HVs were cultured in a UC-MSC-conditioned medium (UC-MSCcm) and a control medium. Flow cytometry was used to detect the surface expression of CD80, CD86, BR3, CD40, PD-1, and HLA-DR on CD19+ B cells and assess the percentage of B cells in early and late apoptosis. An enzyme-linked immunosorbent assay (ELISA) quantified the production of BAFF, IDO, and PGE2 in PBMCs and UC-MSCs. Under UC-MSCcm influence, the percentage and mean fluorescence intensity (MFI) of CD19+BR3+ cells were reduced in both SLE patients and HVs. Regarding the effects of the MSC secretome on B cells in lupus patients, we observed a decrease in CD40 MFI and a reduced percentage of CD19+PD-1+ and CD19+HLA-DR+ cells. In contrast, in the B cells of healthy participants, we found an increased percentage of CD19+CD80+ cells and decreased CD80 MFI, along with a decrease in CD40 MFI and the percentage of CD19+PD-1+ cells. The UC-MSCcm had a minimal effect on B-cell apoptosis. The incubation of patients’ PBMCs with the UC-MSCcm increased PGE2 levels compared to the control medium. This study provides new insights into the impact of the MSC secretome on the key molecules involved in B-cell activation and antigen presentation and survival, potentially guiding the development of future SLE treatments.

## 1. Introduction

Systemic lupus erythematosus (SLE) is a classic autoimmune disease that has attracted significant interest among research teams due to its severity; 400,000 people worldwide receive this diagnosis each year [1]. This chronic autoimmune disease predominantly affects women and is characterized by multiorgan involvement. Common manifestations include mucocutaneous (skin and mucous membrane) symptoms, musculoskeletal issues, hematological abnormalities, renal complications, and immunological alterations. In some cases, patients may also develop neuropsychiatric SLE, affecting the central nervous system. Without appropriate treatment, SLE can be life-threatening and may lead to fatal outcomes. Patients receive treatments, including peroral or intravenous corticosteroids, hydroxychloroquine, and immunosuppressive and target-specific biological drugs, though some may not respond to these therapies. The only approved biological agents for treating these patients are Belimumab (2011) [2], an anti-B-cell activating factor (BAFF) medication, and Anifrolumab, an interferon-α/β receptor 1 (IFNAR1) antagonist (2021) [3]. BAFF is a crucial ligand that promotes the activation, survival, and maturation of autoreactive B cells by binding to three types of receptors: BAFF-R (also known as BR3), BCMA (B-cell maturation antigen), and TACI (transmembrane activator and calcium modulator) [4]. Increased levels of BAFF have been observed in lupus and other autoimmune diseases [5]. A major challenge for researchers is the complex immunopathogenesis of the disease and the intricate communication between immune cells mediated by various membrane-bound and soluble molecules [6]. Despite the involvement of many immune cells, B lymphocytes play a key role in the disease as they secrete autoantibodies and produce other essential inflammatory molecules, such as IL-6, TNF-α, LTa1b2, GM-CSF, and IFN-γ [7]. Additionally, B cells participate in presenting autoantigens by HLA class I and II molecules, driving or amplifying the autoimmune process through cytokine secretion and the expression of co-stimulatory molecules, such as CD80/CD86 and CD40 [8].

Mesenchymal stem cells (MSCs) are an area of research for potential lupus treatments [9]. MSCs are a heterogeneous population of post-embryonic non-hematopoietic stem cells that can be isolated from various sources, including bone marrow, placenta, umbilical cord, adipose tissue, dental pulp, peripheral blood, skin, muscle, and endometrium [10]. This source diversity is crucial for selecting the most appropriate stem cells for specific pathological conditions, such as SLE. Both in vitro and in vivo studies have demonstrated that MSCs from different tissues have immunosuppressive effects on T cells [11], NK cells [12], monocytes, and macrophages [13]. However, fewer studies have focused on the impact of MSCs and their secretome on checkpoint and co-stimulatory molecules on B cells, which are essential for immune responses and tolerance. Corcione’s team has pioneered the study of MSC effects exerted on B-cell surface molecules. In 2006, A. Corcione and colleagues reported that MSCs had no significant impact on B-cell co-stimulatory molecule expression after culturing human MSCs from bone marrow and B cells from healthy donors’ peripheral blood [14]. Most research has concentrated on MSC effects on B-cell proliferation, differentiation, and antibody secretion rather than B-cell molecule expression [15,16,17]. Currently, 19 clinical trials are listed on clinicaltrials.gov investigating MSC treatment for SLE, 4 of which have been completed, and 8 are of unknown status. Recently, attention has shifted to the MSC secretome—the collection of molecules produced by MSCs and found in a conditioned medium—and its potential role in treating various conditions [18,19]. Only one completed clinical study on the MSC secretome and lupus is listed on clinicaltrials.gov, but it lacks detailed results (NCT05921058). This gap highlights the need to advance research on MSCs, their secretome, and B lymphocytes in lupus patients to better understand the paracrine effects of MSCs and this cell-free therapeutic approach.

## 2. Results

### 2.1. Generation of Conditioned Media (MSC Secretome)

#### 2.1.1. Umbilical Cord Mesenchymal Stem Cells (UC-MSCs)

**Figure 1:**
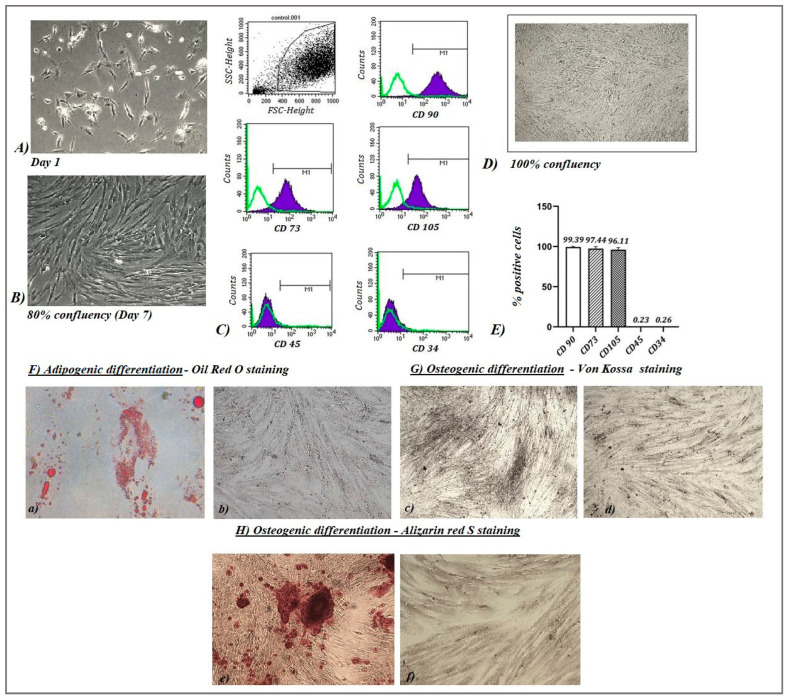
Characterization of the isolated umbilical cord mesenchymal stem cells (UC-MSCs). MSCs isolated from the umbilical cord on day 1 (**A**) show visible fibroblast-like morphology (400×, phase contrast). After reaching 80% confluency (**B**), the conditioned medium was successfully received (400×, phase contrast). Flow cytometric histograms representing the expression of markers CD90, CD73, and CD105, as well as the absence of markers characteristic of hematopoietic lineage, are shown (**C**) together with light microscopic photo (100×) that shows the degree of confluency (100%) of the cells at which the analysis was performed (**D**). The graph represents the mean values of the percentages (Mean ± SD) for each marker examined (data are mean of seven experiments) (**E**). Representative light microscopic images of adipogenic (**F**) and osteogenic (Von Kossa staining (**G**) and Alizarin red S staining (**H**)) differentiated cells are shown. Lowercase letters on the microscopic images represent cells cultured in adipogenic differentiation medium ((**a**) (400×)), osteogenic differentiation medium ((**c**,**e**) (50×)), and in control medium ((**b**,**d**,**f**) (50×)).

MSCs from seven umbilical cord donors were successfully isolated and cultured. The potential of cells from each sample to adhere to the plastic surface of cell culture flasks was microscopically visualized and documented. A microscopic examination shows heterogeneous single-cell populations of UC-MSCs of varying size but with similar fibroblast-like morphology (Figure 1A). A conditioned medium (MSC secretome) from each sample was collected after reaching 80–90% confluency (around day 7) of the cells in each cell culture flask (Figure 1B).

#### 2.1.2. Characterization of UC-MSCs

Following the described minimum criteria of the International Society for Cellular Therapy (ISCT) [20], the isolated UC-MSCs were characterized by flow cytometry analysis after reaching 100% confluency (Figure 1D). We found positive expression of over 90% of the cells of CD90 (99.39 ± 0.82), CD73 (97.44 ± 2.43), and CD105 (96.11 ± 2,55) markers and relatively low to no presence of CD34 (0.26 ± 0.18) and CD45 (0.23 ± 0.22) markers on the cellular surface of UC-MSCs (Figure 1E). The differentiation potential of isolated MSCs from umbilical cords in adipogenic and osteogenic directions was successfully demonstrated by documenting the formed lipid droplets in adipogenically differentiated cells and the presence of calcium deposits in cells that underwent osteogenic differentiation (Figure 1F,G).

### 2.2. Peripheral Blood Mononuclear Cells (PBMCs)

**Figure 2:**
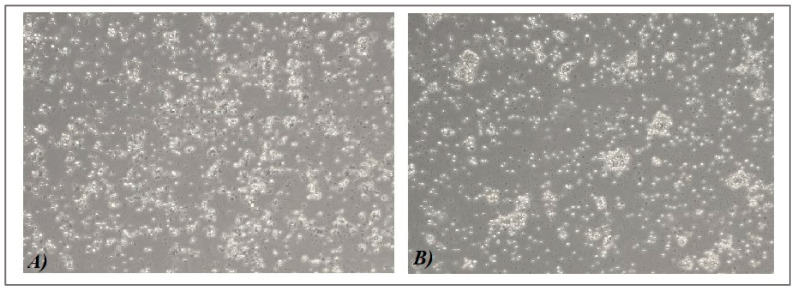
Phase contrast images (100×) of systemic lupus erythematosus (SLE) patients’ peripheral blood mononuclear cells (PBMCs) after 72 h of culture. (**A**) PBMCs, cultured in a control medium and (**B**) PBMCs cultured in a conditioned medium of umbilical cord MSCs (UC-MSCcm), with a significantly higher degree of cell cluster formation.

The average concentration of isolated PBMCs from healthy volunteers (HVs) was 1.42 *×* 10^7^ ± 0.23 (mean ± SD), and from SLE patients, it was 1.28 *×* 10^7^ ± 0.67 (mean ± SD). After 72h of cell culture, phenotypic changes were visible under a light microscope. Larger clusters of SLE-PBMCs were formed when the factors influenced cells in the MSC medium but not under normal culture conditions in the control medium (Figure 2A,B).

### 2.3. Expression of B-Cell Markers Associated with Their Activation, Antigen-Presenting Function, and Survival Is Influenced by the MSC Secretome

#### 2.3.1. Influence of MSC Secretome on CD80, CD86, and BR3 B-Cell Expression

**Figure 3:**
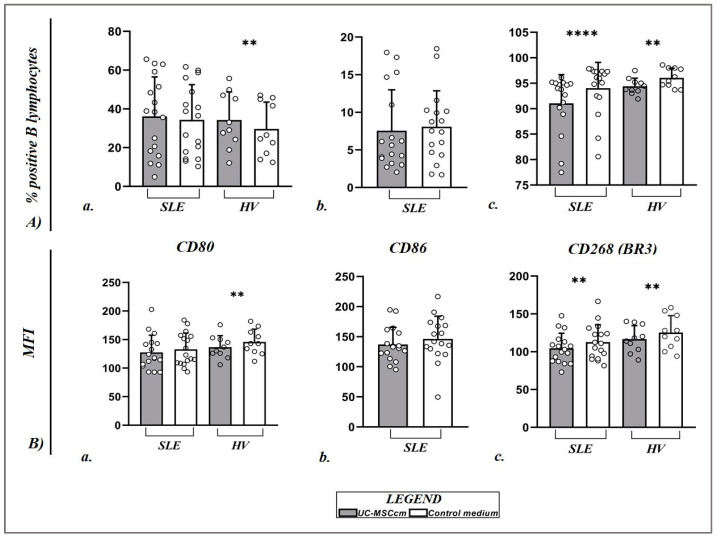
Scatter plots displaying the changes in percentage values (**A**) of CD19+CD80+ B cells ((**A**)(**a**)), CD19+CD86+ B cells ((**A**)(**b**)), and CD19+CD268+ (BR3) B cells ((**A**)(**c**)) and MFI (**B**) of CD80 ((**B**)(**a**)), CD86 ((**B**)(**b**)), and CD268 ((**B**)(**c**)) on the membrane of B lymphocytes of SLE patients (*n* = 17) and healthy volunteers (HVs) (*n* = 10). Data are expressed as mean ± SD, and significant differences are presented after performing the Wilcoxon signed-rank test and Mann–Whitney U test (** *p* ≤ 0.01; **** *p* ≤ 0.0001).

Culturing the isolated PBMCs from the HVs in the conditioned medium resulted in the establishment of a significantly higher percentage of B cells positive for the expression of CD80 under the influence of the MSC secretome (34.26 ± 14.48%) compared to B lymphocytes in the control medium (29.64 ± 13.85%; *p* = 0.0039) (Figure 3A(a)). Regarding the intensity of expression of this molecule (CD80) on the membrane of B cells, we found again in HVs a statistically significant lower level of expression of CD80 in B lymphocytes under the influence of factors released by MSCs (136.71 ± 20.07 vs. 145.93 ± 22.26 *p* = 0.0020) (Figure 3B(a)). In patients with SLE, we found no significant difference either in the percentage of B lymphocytes positive for CD80 or in the level of expression of the molecule. The results of patients with SLE under the influence of the MSC secretome are the following: regarding the percentage of CD19+CD80+ 36.17 ± 20.33% vs. 34.30 ± 18.22%, *p* = 0.1089, and the CD80 mean fluorescence intensity (MFI) 127.66 ± 30.01 vs. 132.92 ± 28.44, *p* = 0.0887, respectively (Figure 3A(a),B(a)).

There was no significant change in CD86+ B lymphocytes under the influence of the UC-MSC-conditioned medium in patients with SLE (7.55 ± 5.44% vs. 8.08 ± 4.78%; *p* = 0.4038) (Figure 3A(b)) or in terms of CD86 MFI (137.08 ± 28.69 vs. 146.32 ± 37.83; *p* = 0.0569) (Figure 3B(b)). In our previous study regarding PBMCs of HVs [21], we reported that exposure to factors secreted by UC-MSCs led to a notable decrease in the MFI of CD86 (145.40 ± 30.32) compared to the expression levels in B lymphocytes from the PBMCs of HVs cultured in a control medium (163.00 ± 28.59). The percentage of CD86+ B lymphocytes cultured in the UC-MSC-conditioned medium (UC-MSCcm) was slightly higher (3.85 ± 3.40) than that of the control group (2.65 ± 2.20), with a statistically significant difference between the two groups [21].

**Figure 4:**
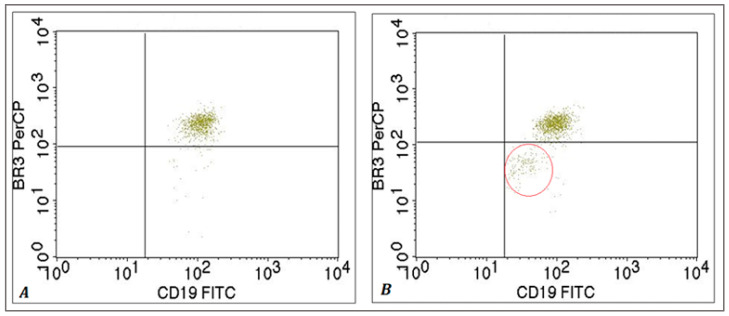
Flow cytometric dot plots of CD19+ B lymphocytes expressing the BR3 receptor from the pool of PBMCs cultured in (**A**) control medium and (**B**) UC-MSCcm. The red represents the formation of a homogeneous population of B lymphocytes with reduced expression of the BR3 receptor, influenced by the secretome of MSCs. A representative patient with SLE is shown in the figure.

We found a statistically lower percentage of BR3+ B lymphocytes under the influence of factors secreted by MSCs compared to the control group B lymphocytes in the HVs (94.40 ± 1.58% vs. 96.07 ± 1.89%; *p* = 0.0020) and patients with SLE (91.06 ± 5.61% vs. 94.05 ± 5.03%; *p* < 0.0001) (Figure 3A(c)). The same trend was observed for the BR3 MFI in HVs (116.78 ± 17.76 vs. 125.43 ± 22.34; *p* = 0.0020) and SLE patients (104.53 ± 19.90 vs. 112.83 ± 22.86; *p* = 0.0032) (Figure 3B(c)). It is noteworthy that after the isolation and culture of B cells from the studied patients and HVs in a conditioned medium of UC-MSCs, we observed the formation of a more homogeneous population of low-expressing BR3-receptor B lymphocytes, which can also be seen from the presented representative for all the experimental setup flow cytometric dot plots (Figure 4).

#### 2.3.2. Influence of MSC Secretome on CD40, CD279 (PD-1), and HLA-DR B-Cell Expression

**Figure 5:**
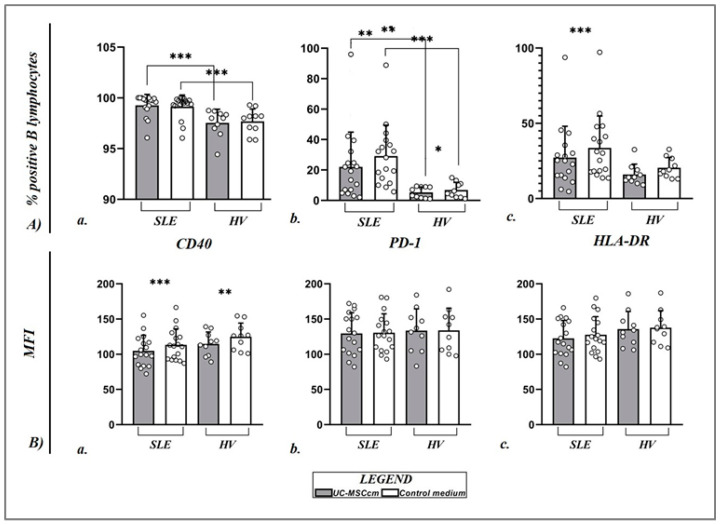
Scatter plots displaying the changes in percentage values (**A**) of CD19+CD40+ B cells ((**A**)(**a**)), CD19+CD279 (PD-1)+ B cells ((**A**)(**b**)), and CD19+HLA-DR+ B cells ((**A**)(**c**)) and MFI (**B**) of CD40 ((**B**)(**a**)), PD-1 ((**B**)(**b**)), and HLA-DR ((**B**)(**c**)) on the membrane of B lymphocytes of SLE patients (*n* = 17) and HVs (*n* = 10). Data are expressed as mean ± SD, and significant differences are presented after performing Wilcoxon test and Mann–Whitney U test (* *p* ≤ 0.05; ** *p* ≤ 0.01; *** *p* ≤ 0.001). Black lines represent significant differences between the two groups of participants.

Data obtained by our team on the percentage of CD40+ B lymphocytes after culturing PBMCs in the conditioned medium of MSCs show a barely detectable change. The percentage of CD40+ B lymphocytes in HVs (97.55 ± 1.33% vs. 97.69 ± 1.21%) and patients with SLE (99.27 ± 1.08% vs. 99.15 ± 1.14%) remained relatively high (Figure 5A(a)). However, the percentage of CD19+CD40+ cells in patients with SLE was significantly higher than that observed in the group of healthy participants after culturing the cells in the control medium (*p* = 0.0010) and the MSC-conditioned medium (*p* = 0.0007). The results are substantially superior concerning the CD40 MFI: the intensity of CD40 expression on B cells under the influence of the conditioned medium of MSCs was significantly decreased compared to the control group of B cells in healthy individuals (114.70 ± 16.91 vs. 124.94 ± 19.23; *p* = 0.0020), as well as in patients with SLE (104.89 ± 22.04 vs. 113.45 ± 22.48; *p* = 0.0008) (Figure 5B(a)).

Under the influence of UC-MSC media, the percentage of CD19+ cells expressing the PD-1 marker was significantly reduced compared to the control media-treated CD19+ cells in HVs (5.28 ± 3.62% vs. 6.87 ± 5.01%; *p*= 0.0273) and patients with SLE (22.07 ± 22.82% vs. 29.14 ± 20.29%; *p* = 0.0011) (Figure 5A(b)). However, again, we did not find a similar trend in the PD-1 MFI in both groups of participants: HVs (133.64 ± 30.74 vs. 135.50 ± 30.05; *p* = 0.6406) and SLE patients (129.65 ± 29.50 vs. 130.54 ± 26.94; *p* = 0.6441) (Figure 5B(b)).

In our study of SLE patients, we observed a notable reduction in the proportion of CD19+ HLA-DR+ cells in the PBMC culture when exposed to the UC-MSCcm (27.24 ± 20.76% vs. 33.63 ± 21.24%; *p* = 0.0005). In the case of healthy individuals, we noted a decreasing pattern (15.93 ± 6.91% vs. 20.44 ± 6.82%; *p* = 0.0527) (Figure 5A(c)). Regarding the intensity of expression of this marker on the B-cell membrane, we did not establish statistical significance, yet the results obtained show a numerically decreased level of expression of the HLA-DR molecule on the B-cell membrane under MSC secretome action in the HVs (135.79 ± 24.87 vs. 137.83 ± 24.15; *p* = 0.3359) and patients diagnosed with SLE (122.57 ± 25.33 vs. 127.72 ± 25.74; *p* = 0.1594) (Figure 5B(c)).

### 2.4. Influence of MSC Secretome on Apoptotic and Necrotic CD19+ B Cells from Peripheral Mononuclear Cells

**Figure 6:**
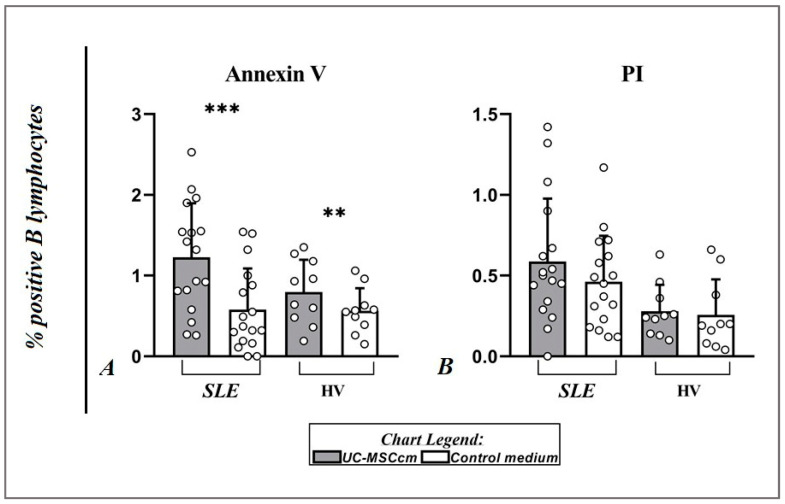
Scatter plots display the percentage value alterations of CD19+Annexin V+ cells (**A**) and CD19+PI+ cells (**B**). Data are expressed as mean ± SD, and significant differences are presented after performing Wilcoxon sign-rank test (** *p* ≤ 0.01; *** *p* ≤ 0.001).

From investigations on the apoptosis of B lymphocytes, we found that the secreted factors in the UC-MSCcm had a feeble influence. However, we observed a significantly higher percentage of Annexin V+ B cells under the influence of MSC factors compared to the control group of B cells in HVs (0.79 ± 0.40% vs. 0.56 ± 0.28%; *p* = 0.0059) and lupus patients (1.23 ± 0.67% vs. 0.65 ± 0.47%; *p* = 0.0007) (Figure 6A). Regarding the results for propidium iodide (PI) positive B cells that we were able to derive after the experiments were performed, we were not able to find a statistical difference in healthy individuals (0.28 ± 0.16% vs. 0.26 ± 0.22%; *p* = 0.5586) and SLE patients (0.59 ± 0.39% vs. 0.46 ± 0.28%; *p* = 0.1324) (Figure 6B). These results, relating to the weak influence of the MSC secretome on B lymphocytes in early and late apoptosis, are also supported by the visible vital cells in Figure 2. Moreover, we demonstrate that the increased percentage of Annexin V+ CD19+ cells positively correlated with the decreased CD40 MFI under the influence of the UC-MSCcm in SLE patients (Spearman’s rho = −0.56, *p* = 0.018).

### 2.5. Influence of MSC Secretome on PGE2 Levels After Culturing PBMCs in UC-MSCcm

**Figure 7:**
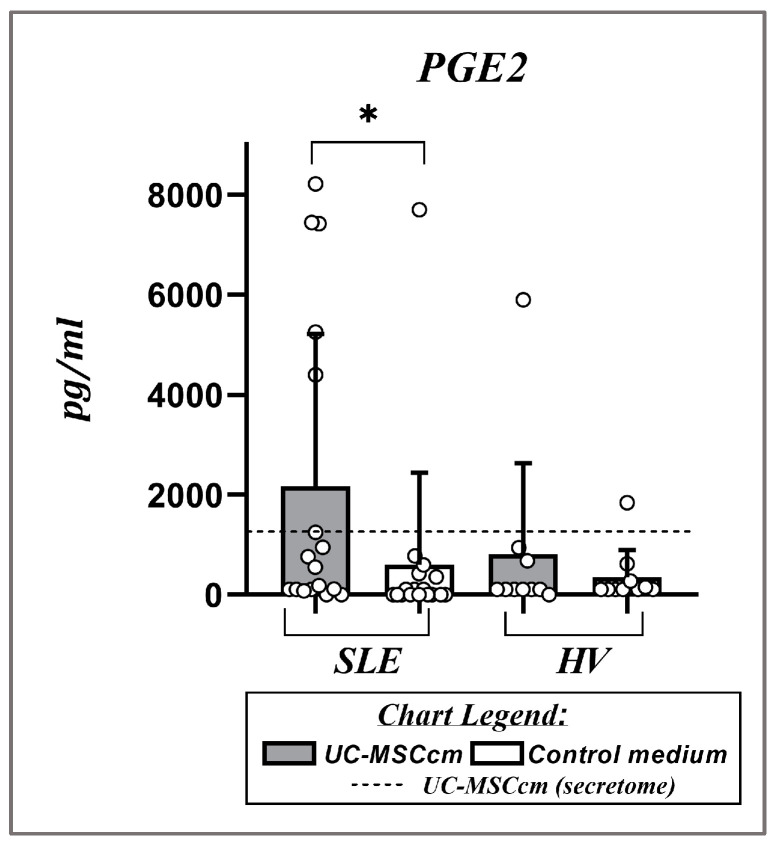
A scatter plot displays the changes in PGE2 levels (pg/mL) after culturing PBMCs in a UC-MSCcm and control medium. Data are expressed as mean ± SD, and significant differences are presented using Wilcoxon sign-rank test (* *p* ≤ 0.05).

Despite our efforts to identify IDO and BAFF in the culture media of PBMC UC-MSCs, neither was detected under the conditions of our experiments. Although we used an enzyme-linked immunosorbent assay (ELISA) with sensitivity parameters optimized for detecting low concentrations of the molecules, the observed BAFF and IDO levels were below the minimum detectable threshold of the commercial ELISA kits. In the UC-MSCcm, the BAFF and IDO levels were also below the minimum detectable threshold of the ELISA method. Regarding PGE2, we observed significantly higher levels of the studied PGE2 after culturing PBMCs in the stem cell medium compared to the control medium in patients with SLE (2173.04 ± 3043.09 pg/mL vs. 597.89 ± 1846.31 pg/mL; *p* = 0.0342), while in HVs, we were unable to confirm this trend with statistical certainty (815.44 ± 1812.92 pg/mL vs. 351.87 ± 547.03 pg/mL; *p* = 0.2730) (Figure 6). We also detected the presence of PGE2 in the stem cell media at the concentration of 1302.13 ± 2073.00 pg/mL, respectively (Figure 7).

## 3. Discussion

The secreted factors from MSCs are mainly known for their anti-inflammatory and immunomodulatory properties, comprising promising biological material for future experimental and clinical research in the autoimmunity field [22]. The diverse effects of the MSC secretome are responsible for its immunomodulatory potential. While a lack of clear information on its impact on B cells hinders the progress of innovative therapies, B lymphocytes are nonetheless a subject of interest due to their widely acknowledged characteristics and significance in autoimmune immunopathogenesis, especially SLE, often referred to as “B-cell disease” [23].

We decided to utilize a conditioned medium instead of the transwell system to study the paracrine action of MSCs on B lymphocytes. The transwell system involves PBMCs and MSCs cultured with a barrier that allows the passage of secretory factors but restricts direct intercellular contact. A conditioned medium was obtained after culturing one cell type (UC-MSCs) to observe its impact on another cell type (B lymphocyte) [24]. Moreover, we preferred to use the conditioned medium to examine how the secretory factors of stem cells affect B cells initially on the grounds that an isolated and well-preserved conditioned medium contains a complete MSC secretome, abundant in biologically active molecules and secretory vesicles [25].

Furthermore, our choice of the in vitro experimental approach was influenced by the aim of the study to explore the paracrine action of “native” unstimulated (chemically or biologically) MSCs. It is important to consider the potential indirect impact of other cells in the culture medium on B lymphocytes, as MSC factors might affect these cells with subsequent changes in the B-cell function. It is crucial to take into consideration that in vivo cells do not function independently from one another [26]. Our team captured microscopic images demonstrating the maintenance of this cell interaction even in in vitro settings. Clusters of non-adherent cells with a greater inclination were observed in the PBMCs of SLE patients cultured in the UC-MSCcm, which we have previously reported for PBMCs from HVs [21].

In our research on the expression profile of CD80/CD86 (B7.1/B7.2) markers on B-cell surfaces, we focused on their crucial involvement in the immune response through their activation and co-stimulatory signals. Professional antigen-presenting cells (APCs), like B lymphocytes, macrophages, and dendritic cells, express B7 molecules and provide the necessary stimulatory signals for T-cell activation and viability by interacting with their T-cell membrane counterpart, CD28. On the other hand, these molecules are considered modulators of immune activation and tolerance of T cells due to the interaction of B7 with CTLA-4 [27]. Despite the similarities between CD80 and CD86, they are encoded by different genes and can have different expression patterns and regulatory roles in immune responses. For example, Suvas et al. showed that CD80 provides a negative signal for the proliferation and secretion of IgG by normal B cells and B cell lymphomas, while CD86 promotes the active state of this type of lymphocytes [28]. We also recognize the importance of this co-stimulatory signal, because without it, T cells will become anergic or undergo apoptosis. Significant results were found only in healthy subjects in terms of an increased percentage of CD19+ CD80+ cells and decreased membrane expression (MFI) of CD80 under the influence of the UC-MSCcm. Our team observed the same results for CD86 in HVs [21]. These results may be explained by the binding of CTLA-4 to B7 and the ability to remove the molecules from the B-cell membrane by processes such as transendocytosis and trogocytosis [29,30]. On the other hand, it is well known that CTLA-4 is expressed mostly by T-regulatory cells, whose differentiation and activation may result from molecules secreted by MSCs [31]. From the experiments performed, we can conclude that the MSC secretome leads to the immune dysfunction of B cells in healthy individuals due to an increased percentage and decreased membrane expression of CD80 and CD86. SLE patients often show altered expression of co-stimulatory molecules, which may account for the limited impact of the MSC secretome [32].

Continuing with the topic of co-stimulatory molecules, we must also highlight the results obtained by our team regarding CD40. The CD40–CD40L interaction is associated with essential processes, such as B-cell activation and proliferation, immunoglobulin class switching and antibody secretion, memory B-cell survival, and suppression of B-cell apoptosis [33]. Previous reports indicate that CD40 signaling drives B cells to an intermediate phenotype between naïve and fully differentiated antibody-secreting cells [34]. Consequently, the observed decrease in CD40 membrane expression resulting from culturing PBMCs from healthy and SLE individuals in the stem cell medium may be linked to a predominance of B cells with a naïve or anergic phenotype. An interesting finding by our team is the negative correlation between the reduced membrane expression of CD40 and the increased percentage of B cells in SLE patients undergoing early apoptosis. These data underscore the importance of CD40 in providing survival signals to B cells [35]. A recent study confirmed the concept of the high expression of CD40, HLA-DR, and IL-21R in the activated naïve B cells of patients with SLE, whose interaction with CD4 T cells contributes to the pathogenesis of the disease [36]. The decrease in the expression level of CD40 and CD19+HLA-DR+ cells could be part of the anti-inflammatory effect of the MSC secretome against B cells.

Data on the influence of MSCs on the expression of HLA-DR on the B-cell membrane are scarce. However, adipose tissue MSCs have been shown to suppress the expression of HLA-DR in activated CD4 and CD8 T lymphocytes [37]. Furthermore, our results, indicating a decreased percentage of CD19+HLA-DR+ B cells, are consistent with the reduced membrane expression of CD40, which stimulates the expression of molecules involved in antigen presentation.

SLE patients frequently exhibit elevated levels of PD-1 on various immune cells, including T- and B cells [38,39]. This upregulation can be a response to chronic antigenic stimulation and inflammation. Patients with SLE included in our study also showed a higher percentage of PD-1 compared with HVs. PD-1 is considered a novel regulator of B-cell activation [40]. Expression of PD-1 increases in response to B-cell activation. However, high levels of PD-1, particularly in chronic inflammatory or autoimmune contexts, might indicate an attempt to control excessive activation or a state of functional exhaustion. In our experiments, the MSC secretome leads to a decreased percentage of PD-1-positive B cells in SLE patients and HVs and can possibly restore more balanced immune responses and reduce autoimmunity.

A reduction in the percentage of BR3+ B cells in parallel with the decreased BR3 MFI in SLE patients and HVs is consistent with the results of Shabgah et al., which showed a decreased percentage of CD19+ BR3+ and CD19+ BCMA+ B cells 12 months after MSC transplantation in patients with rheumatoid arthritis [41]. Contrary to this, another team investigating patients with chronic graft-versus-host disease (GVHD) demonstrated that treatment with MSCs led to an increase in the number of CD27 memory B cells and a decrease in plasma BAFF, associated with the increased expression of BAFF-R in peripheral B cells [42]. Another important point to note is the higher percentage of BR3-positive B cells in healthy individuals compared to those with SLE, which may be explained by the higher plasma levels of BAFF in patients and the potential internalization of the receptor following ligand binding [43]. However, this theory is not supported by our results owing to the absence of detectable levels of BAFF in the culture media from PBMCs and a lack of data obtained regarding plasma levels in patients and HVs. BAFF is associated with stimulating the expression of the gene for CD40, which belongs to the genes directly related to B-cell survival. One possible explanation is that the increased percentage of B cells in apoptosis is due to the decreased membrane expression of CD40 and BR3 [44].

The influence of secreted molecules on the expression of the investigated B-cell markers extends beyond the action of BAFF. With the most significant effect on B lymphocytes are biological molecules such as IDO, PGE2, IL-1RA, CCL-2, CCL5, CCL7, IL-10, TGF-b, Gal-9, IFN-α/β, etc. [45,46]. However, it is difficult to determine which one of them specifically is responsible for the results obtained. One plausible reason for the lack of detectable levels of IDO in the media from stem cells and cultured PBMCs is the absence of an inflammatory signal, such as IFN-gamma, which is associated with the secretion of this enzyme [47]. On the other hand, PGE2 is associated with inhibition of B-cell proliferation and BCR-mediated activation [48], suppressed membrane expression of MHC II [49], and induction of apoptosis [50]. However, PGE2 can also have anti-apoptotic effects depending on the receptor subtype to which it binds and the cellular environment [51]. The overall impact of PGE2 on B cells may vary depending on factors such as the presence of other cytokines and the stage of B-cell development.

## 4. Materials and Methods

### 4.1. Study Subjects, Sample Collection, and Clinical Assessments

A total of 17 patients matching the 2019 EULAR/ACR classification criteria for SLE were enrolled in the study. The selection and clinical evaluation of the subjects were performed in the Rheumatology Clinic of the University Hospital St. Ivan Rilski in Sofia, Bulgaria, over a period of 1 year. Ten HVs were also included in the study. Each subject signed a voluntarily informed consent in accordance with the ethical recommendations of the Helsinki Declaration. Biological material was voluntarily taken from each participant—peripheral venous blood (volume of blood varied between 7 and 12 mL) by vacutainer (VACUTTE^®^ TUBE 3 mL, Greiner Bio-one) for hematology with the anticoagulant, ethylenediaminetetraacetic acid (EDTA). Thereafter, the density gradient centrifugation technique was applied to isolate each patient’s PBMCs from peripheral venous blood. Further information on PBMC cultivation can be found in the Section 4.5. The assessment of disease activity in SLE patients was determined by the Systemic Lupus Erythematosus Disease Activity Index (SLEDAI-2K), and disease activity was defined as SLEDAI-2K ≥ 6. The main inclusion criteria were men and women over the age of 18 with a diagnosis of SLE or newly diagnosed SLE; individuals willing and able to sign informed consent for participation in the study; patients with moderate or high disease activity, defined as SLEDAI-2K ≥ 6, and/or the presence of immunological activity; and increased anti-dsDNA (>25 U/mL) and/or decreased complement components C3 (0.81–1.57 g/L) and C4 (0.13–0.39 g/L). The main exclusion criteria were individuals under the age of 18; previous therapy with the biologic drug (Belimumab) directed against the B-cell activation factor, BAFF; individuals with an active or chronic viral/bacterial infection (including hepatitis B- or C-virus infection, HIV infection, active or latent tuberculosis infection, or other active infectious disease) when the biological material was taken; concomitant malignant disease; and other somatic or mental illnesses or laboratory abnormalities, which could affect the interpretation of the results of the study and, in the opinion of the team, make the patient ineligible to be enrolled in the study. Regarding patient therapy, a total of ten SLE patients were on Hydroxychloroquine (HCQ), and seven of the patients were treated with conventional immunosuppressive drugs. The demographic, clinical, and immunological characteristics of patients with SLE and the HVs are summarized in Table 1.

### 4.2. UC-MSCs: Isolation, Culture, and Conditioned Media Preparation

Human UC-MSCs were isolated from healthy donors (*n* = 7) after obtaining signed written informed consent in accordance with Annex 1-A of the contract for the collection, examination, and storage of MSCs from the umbilical cord tissue of Tissue Bank BulGen, Sofia, Bulgaria. Human umbilical cord tissues were collected under aseptic conditions immediately after the birth of children and the cutting of the umbilical cord. The umbilical cords were delivered in the laboratory in special tubes with a sampling medium (sterile 0.9% NaCl solution) and stored at 4 °C until their processing within 24 h from sampling.

Each umbilical cord was processed following a standard algorithm at work according to the methodology described in our previous publication [21]. Cells were seeded in 10 × 25 cm^2^ (T25; Biologix, Saint Louis, MO, USA) cell culture flasks at 1 × 10^4^ cells/cm^2^ concentrations. The following day, the medium was replaced with fresh medium (6 mL per T25), and the fibroblast morphology of the MSCs was documented using an inverted light microscope (Leica, Wetzlar, Germany). The flasks were stored in an incubator, and the cell culture medium was changed every 48 h until the cells reached 80–90% confluency in each 25 cm^2^ flask. When the cells reached 80–90% confluency and were ready for their secretome to be collected and stored, the cell medium was aspirated and replaced with fresh medium, in which the cells were cultured for an additional 48 h. After this cultivation period, the collected medium, UC-MSCcm, was centrifuged at 876× *g* for 10 min to remove cell debris. The conditioned medium was aliquoted (3 mL in 15 mL tubes) and stored at −80 °C for subsequent experiments as part of the present study.

### 4.3. Phenotyping of UC-MSCs

In order to determine the expression of specific markers for MSCs, cells were washed (CellWash solution, BD Pharmigen, Franklin Lakes, NJ, USA) and labeled with fluorochrome-conjugated antibodies for 15 min in the dark. After labeling, the cells were rewashed and fixed with FIX solution (BD Pharmigen, San Diego, CA, USA). The cell surface markers of UC-MSCs were analyzed using the following antibodies: anti-CD45-FITC/CD34-PE, anti-CD73-PE, anti-CD90-FITC, and anti-CD105-PerCP/Cy5-5 (all from BD Pharmingen, San Diego, CA, USA). The specific fluorescent labeling was analyzed on a FACSCalibur flow cytometer (BD, San Diego, CA, USA) using BD CellQuest Pro software Version 6.0 (BD, San Diego, CA, USA).

### 4.4. Investigation of the Differentiation Potential of the Isolated MSCs

Cell cultures of UC-MSCs (passage 2) reaching above 80% confluency were trypsinized, seeded in 6-well plates (Greiner bio-one, Austria) at a concentration of 5 x 10^4^ cells/cm^2^, and cultured under standard cell culture conditions in a specific medium inducing osteogenic or adipogenic differentiation, and successful differentiation was determined using Alizarin red S, Von Kossa, and Oil Red O staining methods. For a comprehensive description of the MSC differentiation procedure, please refer to our previous publication [52]. The osteogenic and adipogenic induction medium was replaced every 48–72 h for 21 days. For the negative control, cells were cultured in a regular medium of Dulbecco′s Modified Eagle′s Medium/Nutrient Mixture F-12 (DMEM/F12), 10% fetal bovine serum (FBS), and 1% penicillin–streptomycin/amphotericin B mix (Pen/Strep/Amph B Mix).

### 4.5. Isolation and Culture of PBMCs

PBMCs from healthy volunteers and SLE patients were isolated by density gradient centrifugation using Ficoll Paque™ PLUS (GE Healthcare BioScienc, Chicago, IL, USA). Isolated cells were cultured for 72 h at a concentration of 1 × 10^6^ cells/well in a six-well plate (9.6 cm^2^, Greiner bio-one, Kremsmünster, Austria) in a UC-MSCcm and 1 × 10^6^ cells/well in a regular medium under standard cell culture conditions. After incubation, the cells were centrifuged at 350× *g* for 10 min. The supernatant above the pellet was separated for subsequent immunoenzyme methods, and the cells were prepared for the flow cytometric analysis of B lymphocytes.

### 4.6. Phenotyping of CD19^+^ B Cells

PBMCs at a concentration of 1 × 10^6^ cells (cultured in the UC-MSCcm and regular medium) were washed twice in BD cell wash and tested for the surface expression of anti-CD19 FITC (Biolegend, San Diego, CA, USA); anti-CD80 PE (BD Pharmingen, San Diego, CA, USA); anti-CD86 APC (BD Pharmingen, San Diego, CA, USA); anti-CD268 (BR3) Per CP/Cy5.5 (Biolegend, USA); anti-CD40 PE (BD Pharmingen, San Diego, CA, USA); anti-HLA-DR Per CP (BD Pharmingen, USA); and anti-CD 279 (PD-1) APC (BD Pharmingen, San Diego, CA, USA). The cells were further processed in line with the manufacturer’s instructions, fixed with CellFix (BD, San Diego, CA, USA), and counted on a FACSCalibur flow cytometer (BD, San Diego, CA, USA) and using BD CellQuest software Version 6.0. Data are presented as percentages and MFIs.

### 4.7. Apoptosis

In order to evaluate the percentage of B cells in early and late apoptosis stages, cells were tested for the surface expression of phosphatidylserine and PI staining using an Annexin V-FITC Apoptosis Detection Kit (BD Pharmingen, USA). The test was performed with a modification involving two separate tubes for each experiment. One tube contained anti-Human CD19-PerCP (BioLegend, USA) and Annexin V-FITC, while the other tube contained CD19-FITC (BioLegend, USA) and PI-PE. All subsequent steps were carried out according to the manufacturer’s instructions. The acquisition and analysis of the obtained data were performed using a FACSCalibur flow cytometer (BD, USA) and BD Cell Quest software.

### 4.8. Enzyme-Linked Immunosorbent Assay (ELISA)

The conditioned media after culturing PBMCs from both experimental groups (the UC-MSCcm and the control (regular) medium) were tested for the secretion of BAFF/BLYS (Abcam, Cambridge Biomedical Campus, Cambridge, UK), IDO (R&D System, Minneapolis, MN, USA), and PGE2 (R&D System, Minneapolis, MN, USA), according to the manufacturer’s instructions.

### 4.9. Statistical Analysis

The graphic design of the figures and the statistical analysis of raw data were performed using GraphPad Prism version 8.0 and SPSS version 27.0. Descriptive statistics were used to describe the demographic and clinical characteristics, as well as present data on immunological indicators for study patients and HVs. The results are presented as mean ± SD. The normality of data distribution was assessed using the Shapiro–Wilk test and Kolmogorov–Smirnov test to determine the appropriate statistical tests for comparing samples (parametric or non-parametric). Due to the relatively small number of participants and non-normal data distribution, the non-parametric Wilcoxon test for related samples and the Mann–Whitney U test for non-related samples were applied. Spearman’s correlation analysis was used to examine relationships for variables with non-normal distributions. The level of statistical significance was set at *p* ≤ 0.05.

## 5. Conclusions

The interaction between MSCs and B cells represents a crucial aspect of the immune regulatory network with significant implications for health and disease. These interactions are complex and context-dependent, influenced by factors such as the inflammatory environment and the stage of B-cell development. This study demonstrates that MSC-secreted factors can modulate the expression of key receptors and molecules on B cells, such as BAFF-R, and impact B-cell survival and function. Future studies should provide valuable insights into mechanisms through which MSCs and their secretome contribute to this immune regulation, and this combined knowledge could enhance therapeutic strategies for autoimmune diseases and other conditions involving dysregulated B-cell activity.

## Figures and Tables

**Table 1:** Demographic, clinical, and immunological characteristics of patients with systemic lupus erythematosus (SLE) and healthy volunteers (HVs).

		SLE		HV
N		17		10
Age (Years)		42.71 ± 8.28 ^1^		33.5 ± 9.10 ^1^
Gender				
Male (%)		2 (12)		5 (50)
Female (%)		15 (88)		5 (50)
Disease duration (Years)		6.82 ± 7.92 ^1^		-
ANA n (titer)	n (immunofluorescence pattern (ICAP))	13 (≥1:1280) 3 (≥1:320) 1 (1:160)	11 (AC-1) 7 (AC-4) 3 (AC-5) 2 (AC-21-like)	-
Anti-dsDNA (IU/mL)		65.65 ± 68.54 ^1^		-
C3 (g/L)		1.22 ± 0.52 ^1^		-
C4 (g/L)		0.22 ± 0.07 ^1^		-
ESR (mm/h)		15.76 ± 20.66 ^1^		-
CRP (mg/L)		9.42 ± 19.54 ^1^		-
SLEDAI-2K		10.88 ± 6.95 ^1^		-
Clinical manifestations n (%)				-
Musculoskeletal manifestations		15 (48)		-
Mucocutaneous manifestations		10 (32)		-
Hematological abnormalities		2 (7)		-
Renal involvement		2 (7)		-
Neuropsychiatric lupus		2 (6)		-

^1^ mean ± SD; Abbreviations:; ANA (anti-nuclear antibodies); ICAP (International Consensus on ANA Patterns); dsDNA (double-stranded DNA); ESR (erythrocyte sedimentation rate); CRP (C-reactive protein); SLEDAI-2K (Systemic Lupus Erythematosus Disease Activity Index).

## Data Availability

Data are contained within the article.
